# Comparative Study of Predictive Models for the Detection of Patients at High Risk of Inadequate Colonic Cleansing

**DOI:** 10.3390/jpm14010102

**Published:** 2024-01-17

**Authors:** Antonio Z. Gimeno-García, Davinia Sacramento-Luis, Marta Cámara-Suárez, María Díaz-Beunza, Rosa Delgado-Martín, Ana T. Cubas-Cubas, María S. Gámez-Chávez, Lucía Pinzón, Domingo Hernández-Negrín, Alejandro Jiménez, Carlos González-Alayón, Raquel de la Barreda, Manuel Hernández-Guerra, David Nicolás-Pérez

**Affiliations:** 1 Gastroenterology Department, Hospital Universitario de Canarias, Instituto Universitario de Tecnologías Biomédicas (ITB) & Centro de Investigación Biomédica de Canarias (CIBICAN), 38320 Santa Cruz de Tenerife, Spain; dsaclui@gobiernodecanarias.org (D.S.-L.); alu0100973587@ull.edu.es (M.C.-S.); alu0101048932@ull.edu.es (M.D.-B.); rdelmarv@gobiernodecanarias.org (R.D.-M.); acubcub@gobiernodecanarias.org (A.T.C.-C.); mgamcha@gobiernodecanarias.org (M.S.G.-C.); ipinuri@gobiernodecanarias.org (L.P.); dherneg@gobiernodecanarias.org (D.H.-N.); alu0100380437@ull.edu.es (C.G.-A.); rbarheu@gobiernodecanarias.org (R.d.l.B.); mhernand@ull.edu.es (M.H.-G.); dnicper@gobiernodecanarias.org (D.N.-P.); 2 Internal Medicine Department, Universidad de La Laguna, 38320 Santa Cruz de Tenerife, Spain; 3 Research Unit, Hospital Universitario de Canarias, 38320 Tenerife, Spain; ajimsos@gobiernodecanarias.org

**Keywords:** colonoscopy, Boston colonic preparation scale, predictive models, colon cleansing quality, high-risk population

## Abstract

**Background:** Various predictive models have been published to identify outpatients with inadequate colonic cleansing who may benefit from intensified preparations to improve colonoscopy quality. The main objective of this study was to compare the accuracy of three predictive models for identifying poor bowel preparation in outpatients undergoing colonoscopy. **Methods:** This cross-sectional study included patients scheduled for outpatient colonoscopy over a 3-month period. We evaluated and compared three predictive models (Models 1–3). The quality of colonic cleansing was assessed using the Boston Bowel Preparation Scale. We calculated the area under the curve (AUC) and the corresponding 95% confidence interval for each model. Additionally, we performed simple and multiple logistic regression analyses to identify variables associated with inadequate colonic cleansing and developed a new model. **Results:** A total of 649 consecutive patients were included in the study, of whom 84.3% had adequate colonic cleansing quality. The AUCs of Model 1 (AUC = 0.67, 95% CI [0.63–0.70]) and Model 2 (AUC = 0.62, 95% CI [0.58–0.66]) were significantly higher than that of Model 3 (AUC = 0.54, 95% CI [0.50–0.58]; *p* < 0.001). Moreover, Model 1 outperformed Model 2 (*p* = 0.013). However, the new model did not demonstrate improved accuracy compared to the older models (AUC = 0.671). **Conclusions:** Among the three compared models, Model 1 showed the highest accuracy for predicting poor bowel preparation in outpatients undergoing colonoscopy and could be useful in clinical practice to decrease the percentage of inadequately prepared patients.

## 1. Introduction

Colonoscopy is the gold standard for detecting colorectal cancer (CRC), and its use in screening populations allows for the detection of early neoplastic lesions (colorectal adenomas and early CRC). Prospective cohort studies and case–control studies have shown a reduction in CRC incidence and CRC-associated mortality [1]. Quality criteria have been proposed to improve the technique’s efficiency, including appropriate indications and adherence to surveillance intervals recommended either after the removal of precancerous lesions or in inflammatory bowel disease [2]. Two critical quality indicators include the rate of cecal intubation and the percentage of detected neoplastic lesions, both of which are linked to sufficient colonic cleansing [2,3]. Inadequate bowel preparation negatively affects colonoscopy efficiency, increasing costs due to the need for repeat examinations, delaying the diagnosis of malignant or premalignant lesions, reducing the detection rate of these lesions, prolonging the procedure, and likely increasing risks for patients [4]. The rate of inadequate colonoscopies in endoscopy units varies according to studies, ranging from 6.8% to 33% [5,6]. However, the European Society of Gastrointestinal Endoscopy (ESGE) and the American Society of Gastrointestinal Endoscopy (ASGE) consider a percentage between 10% and 15% of colonoscopies with inadequate bowel cleansing to be acceptable [3,4]. Inadequate colon cleansing may result from a lack of effectiveness in the cleansing protocol (colon preparation solution, timing, diet…) or by non-compliance with preparation instructions, whether deliberate or unintentional. Each of these factors is linked to variables dependent on both the patient and the personnel providing information to the patient. Several studies have examined factors that can predict insufficient bowel cleansing [6,7,8,9]. Understanding these factors is crucial, as the cleansing strategy can be tailored based on the characteristics of each patient and the environment in which endoscopic activity takes place. This is of importance since some societies have recommended additional bowel preparation in “hard to prepare” patients [10,11].

To date, three predictive models of poor bowel preparation using a validated cleansing scale as the reference have been reported in outpatients (Supplementary Materials File S1) [7,8,9], with the diagnostic yield varying across the studies (area under the curve from 0.62 to 0.77). The main objective of the predictive models was to detect those patients who benefit most from additional bowel preparation, with the purpose of reducing inadequate bowel preparation rates and minimizing the need for repeated examinations.

Currently, no prospective studies have compared these predictive models, such that, it is unknown which one has greater potential for use in routine clinical practice. The objectives of the present study were to compare these models in an independent cohort of patients, to externally validate them in a separate cohort of consecutive patients and to design a new and improved predictive model.

## 2. Materials and Methods

### 2.1. Patients

The investigation took place at the Endoscopy Unit of the University Hospital of the Canary Islands, a tertiary referral hospital that provides health care to 450,000 residents in the northern region of Tenerife Island. Our endoscopy unit conducts approximately 6000 outpatient colonoscopies each year, with roughly 3000 scheduled for the morning. In this study, individuals undergoing morning outpatient colonoscopies from January 2023 to March 2023 were eligible for inclusion to facilitate the development of the predictive model. The exclusion criteria were as follows: ileus, intestinal obstruction, megacolon, inadequately managed hypertension (systolic blood pressure > 180 and diastolic blood pressure > 100), congestive heart failure, acute liver failure, end-stage renal disease (dialysis or predialysis), New York Heart Association class III–IV, pregnancy, breastfeeding, diagnosis of phenylketonuria, diagnosis of glucose-6-phosphate dehydrogenase deficiency, cognitive impairment with difficulty in taking the preparation, prior inclusion, ingestion of <75% of the cleansing preparation, subtotal colectomy, incomplete colonoscopy, and declining to sign the informed consent document.

**Procedures before the colonoscopy**

Two participating nurses explained the study’s objective to the patients, obtained their informed consent, and administered a questionnaire addressing their medical history and details related to the consumption of the cleansing preparation. All patients were given written instructions on the cleaning protocol, which recommended the use of adjuncts to improve palatability in case of poor tolerance. This intervention aimed to reduce poor tolerance to the preparation. A phone call was made two weeks prior to the colonoscopy to inform patients about the procedure, remind them how to correctly perform the cleansing preparation, and address any doubts, as is routinely done in our digestive endoscopy unit. This intervention aimed to minimize noncompliance. Every participant was provided with both verbal and written guidance, which outlined a prescribed low-fiber diet to be followed on the day preceding the procedure. The patients received a split-dose preparation, beginning the last dose 5 h before the colonoscopy appointment. Any of the following colon preparations routinely used at the hospital were indicated by the requesting physician: Casenglicol^®^ (polyethylene glycol) (Casen Recordati S.L., Utebo (Zaragoza. Spain), Moviprep^®^ (polyethylene glycol plus ascorbic acid) (Norgine BV, Amsterdam, The Netherlands), Citrafleet^®^ (sodium picosulfate, magnesium oxide, citric acid) (Casen Recordati S.L., Utebo (Zaragoza. Spain), and Pleinvue^®^ (macrogol 3350, sodium ascorbate, anhydrous sodium sulfate, ascorbic acid, sodium chloride, and potassium chloride) (Norgine BV, Amsterdam, The Netherlands).

**Procedures the day of the colonoscopy**

Colonoscopies were scheduled every 30 min from 10:00 a.m. to 13:30 a.m. A questionnaire was given to the patients. Subsequently, the colonoscopies were performed by six endoscopists, and the quality of cleansing was scored using the Boston Bowel Preparation Scale (BBPS) [12]. This scale rates each segment of the colon from 0 to 3 points, resulting in a minimum score of 0 points and a maximum score of 9 points. A cleanliness score below 2 points in any segment of the colon is considered suboptimal. The BBPS is routinely used in our endoscopy unit [9]. The endoscopists filled out the data sheet concerning the quality assessment and the number and size of polyps detected by segment. Approval for the study was obtained from the Research Ethics Committee of the University Hospital of the Canary Islands (ethical approval number CHUC_2022_87), and the study was registered at Clinicaltrials.gov (NCT05871801).

**Predictive models**

The current manuscript tested three reported predictive models that utilized the Boston Bowel Preparation Scale (BBPS) as a gold standard. In the first model by Gimeno et al., 667 consecutive patients were included in the model’s developing cohort and 409 in the derivation cohort. Variables with a *p*-value ≤ 0.1 in the simple regression analysis were included in the multiple regression analysis. Only statistically significant variables were used to construct the predictive model. The four variables included in the model are detailed in the Supplementary Materials File S1. The variable with the highest Wald coefficient received a score of 4 points, and the others were scored proportionally. The original study selected a score of 1.225 points as the optimal cut-off to differentiate between adequate and inadequate bowel cleansing. In the model by Dik et al. [8] 1996 consecutive colonoscopies were performed on inpatients and outpatients. Two-thirds of the cases were used to build the model, and the remaining third to validate it. A simple and multiple logistic regression analysis was conducted to identify independent predictors of poor bowel preparation (see Supplementary Materials file S1). Each variable’s value in the model was assigned based on the regression coefficients, ranging from 1 point to 3 points. The optimal cut-off in this case to differentiate between adequate and inadequate bowel preparation was set at ≥2 points. Finally, Beger et al. developed another predictive model that prospectively included 561 consecutive patients. Internal validation was conducted. A simple and multiple logistic regression analysis, similar to the other studies, was conducted. In this case, the odds ratio derived from the β-coefficients was used to assign the score for each variable in the model (see Supplementary Materials file S1). The optimal cut-off in this case was set at ≥2 points.

### 2.2. Statistical Analysis

Results for continuous variables are expressed as medians and 25th–75th percentiles. Categorical variables are expressed as frequencies and percentages. The diagnostic accuracy for inadequate colonic cleansing of the 3 predictive models (Gimeno et al., Model 1; Dik et al., Model 2; and Berger et al., Model 3) was compared using the area under the ROC curve (AUC) of each model. Concerning the sample size, an interim analysis involving 574 patients was conducted, revealing statistically significant differences in the AUC between Model 1 and Model 3, as well as between Model 2 and Model 3. A non-significant trend was observed when comparing Model 1 and Model 2 (*p* = 0.061). Consequently, an additional 75 patients were included, bringing the total to 649 patients. Simple and multiple logistic regression analyses were carried out in order to identify the variables associated with inadequate bowel preparation. Results are expressed as risk ratios and 95% confidence intervals (CIs). All collected variables were included in the simple logistic regression analysis. Bowel cleansing quality (defined as BBPS ≥ 2 in each segment) was the dependent variable, and variables with a *p* value < 0.05 in the simple logistic regression analysis were included in the multiple logistic regression analysis. A novel predictive score was developed by considering variables that demonstrated statistical significance in the multivariate analysis. The maximum value was assigned to the total number of significant variables identified in the logistic regression analysis, and this value was allocated to the variable with the highest Wald coefficient. The remaining values were calculated proportionally. The area under the curve (AUC) was utilized to determine the optimal cutoff for predicting inadequate cleansing. Sensitivity, specificity, positive predictive value (PPV), negative predictive value (NPV), positive likelihood ratio (LR+), and negative likelihood ratio (LR−) were computed for the identified optimal cutoff. Statistical significance was defined as *p* values less than 0.05. The data were subjected to analysis with the Statistical Package for Social Sciences v. 25.0 (Armonk, NY, USA: IBM Corp.) and MedCalc^®^ Statistical Software version 20.106 (MedCalc Software, Ltd., Ostend, Belgium; https://www.medcalc.org; accessed on 1 September 2023).

## 3. Results

**Patient characteristics**

**Figure 1:**
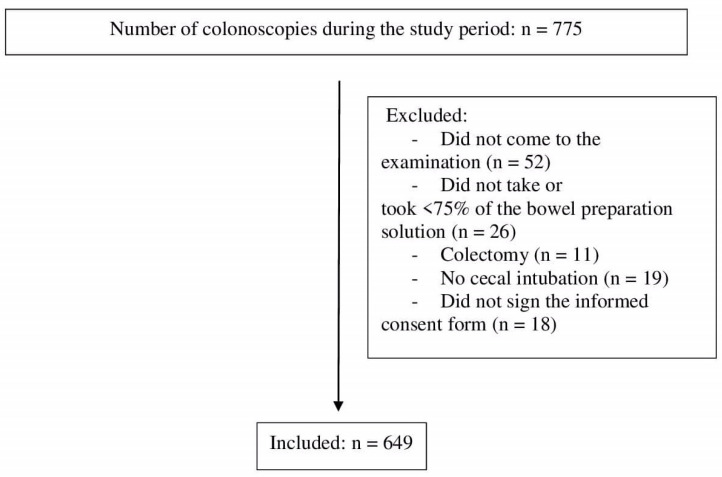
Flow chart.

During the study period, 775 patients underwent a morning colonoscopy, of whom 649 were finally included (Figure 1). A total of 333 (51.3%) were men, and 316 (48.7%) were women. The median age was 61 years (range, 18–90 years). Half of the patients (49%) had primary education, and the median body mass index was 27.51 kg/m^2^. Indications for colonoscopy are shown in Table 1. A quarter (*n* = 167, 25.7%) of the patients had some comorbidity, which was diabetes in most cases (*n* = 153, 23.6%). Approximately one-fifth of the patients suffered from constipation (*n* = 144, 22.2%), 43 patients (6.6%) had an Eastern Cooperative Oncology Group (ECOG) score of ≥2 points, and 42 patients (6.6%) had an American Society of Anesthesiologists (ASA) physical status classification system score of ≥3 points. A total of 213 patients (32.8%) had a history of abdominal or pelvic surgery. In total, 48 patients (7.4%) were on tricyclic antidepressants, 31 (4.8%) were on neuroleptics, and 15 (2.3%) were on opioids. Overall, 348 participants (53.6%) took 2 L of polyethylene glycol (PEG) plus ascorbic acid (Asc), 142 (21.9%) took sodium picosulfate plus magnesium citrate plus citric acid, 104 participants (16%) took 1 L of PEG plus Asc, and 55 participants (8.5%) took 4 L of PEG.

The median score for bowel cleansing, as assessed by the BBPS, was 6 points (with a range of 0–9 points). In comparison to the transverse colon (10.5%) and distal colon (9.1%), the proximal colon exhibited the lowest bowel cleansing scores, with inadequate preparation observed in 88 patients (13.7%). No significant differences were found regarding neoplastic lesions between patients with and without adequate bowel cleansing (36.2% vs. 30.7%, OR 1.29, 95% CI [0.81–2.02], *p* = 0.29). The average time elapsed between the final dose of preparation and the colonoscopy was 3.51 ± 2.04 h.

**Comparison of the three models**

**Figure 2:**
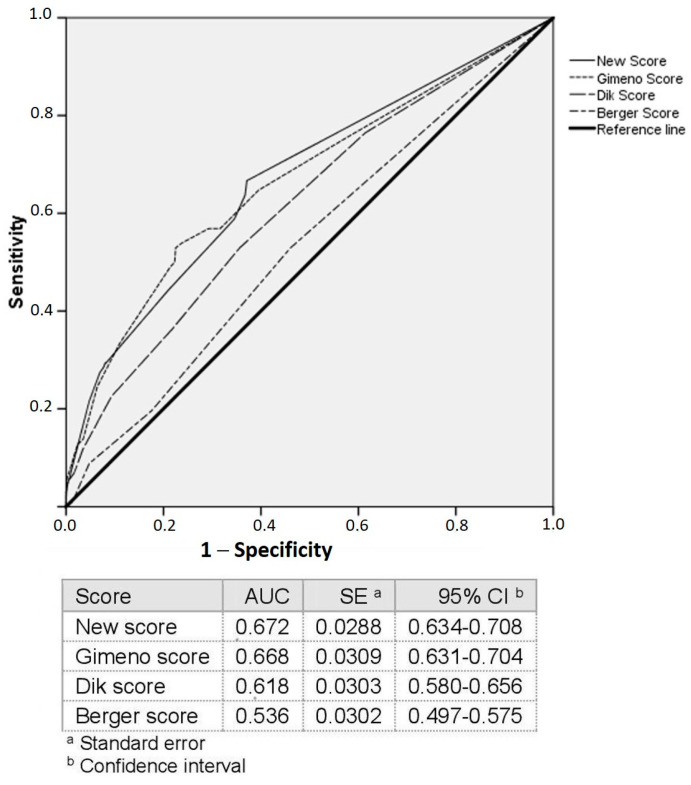
ROC curves.

As shown in Figure 2, Model 1 (AUC = 0.67, 95% CI [0.63–0.70]) and Model 2 (AUC = 0.62, 95% CI [0.58–0.66]) were significantly more accurate than Model 3 (AUC = 0.54, 95% CI [0.50–0.58]) (*p* < 0.001). Model 1 was also more accurate than Model 2 (AUC = 0.67, 95% CI [0.63–0.70]) vs. AUC = 0.62, 95% CI [0.58–0.66], *p* = 0.013). Table 2 shows a comparison of the AUCs of the three models.

**Simple and multiple logistic regression analysis**

In the simple logistic regression analysis, being elderly, having comorbidities, renal failure, stroke, or diabetes mellitus, taking opioids, being on neuroleptics, having a family history of CRC, having a low educational level, and having a low ECOG status were significantly associated with poor bowel cleansing (Supplementary Table S1).

In the multiple logistic regression analysis, renal failure, ECOG performance status, diabetes mellitus, and constipation were independently associated with poor bowel cleansing (Table 3).

**New predictive model**

The independently associated variables were used to build a new predictive model (Supplementary Table S2). As shown in Table 3, suffering from diabetes mellitus exhibited the highest Wald coefficient (14.901), followed by constipation (12.748). Therefore, diabetes mellitus was assigned the highest score, and the remaining scores were proportionally calculated accordingly. The AUC of the new model was 0.67 (95% CI [0.634–0.708]). This model was more accurate than Model 2 (*p* = 0.009) and Model 3 (*p* < 0.001) but similar to Model 1 (Figure 2, Table 2). Table 4 shows the statistical performance of the new predictive model in the total colon and by segment.

## 4. Discussion

We found that the model reported by Gimeno et al. [9] was significantly more accurate for predicting poor bowel cleansing than the other two models. However, the overall predictive ability of all three models was modest. Furthermore, the new model we developed did not show greater accuracy than the previous models.

To our knowledge, this is the first prospective study comparing predictive models designed to assess bowel cleansing in outpatients. Only one other study compared two predictive models, but it was conducted in a retrospective manner [13]. In that study, Afecto et al. compared the predictive scores reported by Dik et al. [8] and Gimeno et al. [9] at a tertiary care center. They included a total of 514 patients, of whom 85.8% had proper bowel cleansing according to the BBPS. The accuracy was comparable between both models, with an AUC of 0.62. The accuracies found in our study were similar, but the predictive score reported by Gimeno et al. was significantly better than the other two.

Nevertheless, we acknowledge that there is room for improvement in the performance of the current predictive models. This study underscores their relatively modest predictive ability for bowel cleansing. Previous studies have consistently demonstrated fair to moderate accuracy. However, they have generally exhibited a commendable NPV, suggesting the effective identification of patients with proper bowel cleansing. To enhance bowel cleansing rates, there is a need for refining these scores. Although we attempted to improve the accuracy of the existing models by creating a new model, including variables used in the previous models, unfortunately, the new model failed to show improvement upon the previous models. The rationale for designing a new predictive model, considering the variables used in the published models, is that all these models did not encompass exactly the same variables or the same number of variables. This fact makes the present work a more comprehensive study, including more variables potentially associated with inadequate colon cleansing. The failure to improve upon previous models lends itself to various interpretations; one of them is that these predictive models no longer have further room for improvement. However, the existence of variables associated with inadequate cleansing cannot be excluded, which have not yet been investigated. However, these models have already included variables found to be associated with poor bowel cleansing in large studies [6,14]. Another explanation is that the predictive models have been designed to identify patients who would benefit most from additional bowel preparation. However, different factors are involved in the quality of bowel cleansing. Factors related to adherence to the bowel preparation protocol, deliberated or not, and tolerance to the cleansing solution can be targeted by educational strategies [15]. Factors associated with deliberate non-compliance include a lack of willingness to adhere to the cleansing protocol, a belief that the cleansing protocol may pose health issues (for example, fasting for diabetic patients), or failure to adhere to the time interval between the intake of the cleansing preparation and the colonoscopy procedure (for example, in patients residing far from the hospital) [16]. Unintentional non-compliance may be motivated by a lack of understanding of the cleansing instructions, either due to the cultural level of the patients or the inadequate drafting and explanation of the instructions. Other factors include poor tolerance of the bowel solution, often stemming from its unpleasant taste or high volume. In such instances, it is advisable to choose low-volume bowel preparations or those with more appealing flavors. Some helpful tricks, such as chewing gum during bowel preparation, consuming beverages like orange or pineapple juice, or the use of Coca-Cola as a diluent for the cleansing solution, may enhance the likelihood of successfully ingesting the entire dose of the bowel solution [16]. Other factors are related to the lack of efficacy of the bowel solution, primarily associated with low bowel motility, such as factors leading to constipation, like abdominal surgeries, comorbidities (for example, diabetes mellitus), or being on treatment with opioids or tricyclic antidepressants. Patients with these factors could benefit from changes in the bowel cleansing protocol, such as increasing the volume of bowel preparation [16]. Precisely, the predictive models have been designed to identify patients who would benefit most from additional bowel preparation. With all this evidence, in patients undergoing their first colonoscopy, the most effective strategy to ensure adequate bowel cleansing is likely a multitarget approach, focusing on compliance with instructions, improving tolerance to bowel preparation, and possibly increasing the quantity of solution in the presence of risk factors for poor bowel cleansing. For patients who had a previous colonoscopy with inadequate bowel preparation, it is important to investigate the potential reasons, primarily lack of adherence to preparation instructions, intolerance, or lack of efficacy, and then tailor an appropriate strategy accordingly [16].

In the present study, efforts were made to ensure adherence and tolerance. In addition to providing written and oral instructions, patients were also contacted by phone 1–2 weeks before the examination by a group of trained nurses to ensure adherence and tolerability.

In the study by Afecto et al. [13], the ASA score was the only variable independently associated with poor bowel cleansing. The authors suggested that the ASA score could be a more parsimonious triage tool in facilities attending to more complex patients, such as tertiary care centers, as it provides similar accuracy with fewer variables, making it easier to use. In our study, however, the ASA score was not independently associated with poor bowel preparation, although the lower complexity of the patients in our study compared to the Portuguese study could have influenced this result (ASA 3–4: 6.6% vs. 24.1%, respectively). In our new predictive model, suffering from renal failure, diabetes mellitus, constipation, and ECOG score were the variables independently associated with inadequate bowel cleansing, with diabetes mellitus being the one with the highest Wald coefficient. These variables have been found to be associated in the other models with the exception of ECOG score. However, the ECOG score is a performance status scale and is likely associated with the degree of comorbidity and sedentary behavior, making its correlation with inadequate colonic hygiene in patients not surprising. Therefore, given that all the variables included in the new model are directly or indirectly linked to inadequate bowel preparation, it is not surprising that the model does not substantially improve upon the previous models.

One interesting finding is the lower accuracy of the models found in this study compared with that in the original studies. These results are in agreement with the Portuguese study. They hypothesized that different characteristics of the samples, especially regarding comorbidities and indications, could have influenced their results. In our previous study, we did not calculate the ASA score. However, the results of the present study are similar to those of the Portuguese study, and the overall ASA score was lower. The study by Afecto et al. [13] also suggested that indications could be another source of heterogeneity. Certainly, the indications for colonoscopy in our previous study and the present study are different from those in the studies by Dik et al., Berger et al., and Afecto et al. [7,8,13]. In Spain, there is a national CRC screening program that has increased the proportion of colonoscopies performed, accounting for 30% of the examinations in our endoscopy unit. The second most common indication is postpolypectomy surveillance, which is in line with other Spanish endoscopy units and is partly a consequence of CRC screening [17]. Subjects in screening populations frequently have few comorbidities and high rates of adequate bowel cleansing [18]. The indications in the other studies were mainly symptoms, inflammatory bowel disease, and endoscopic treatment [7,8,13].

The main strength of this study is that it is the first prospective study to compare three different predictive models in an outpatient setting outside of the original studies in which they were designed. However, we are aware of the limitations of the study, mainly stemming from it being conducted at a single center. As previously suggested, the results could differ depending on the complexity (e.g., comorbidities, medications) of the reference population and the type of facility (tertiary care hospital, community hospital). Although multicenter studies may provide better generalizability, published studies, including ours, have shown that the current systems could be more beneficial for excluding poor bowel preparation than for confirming poor bowel preparation.

In conclusion, the present study demonstrates that the current models may be useful in detecting patients with a high probability of adequate bowel preparation and could be useful in clinical practice, especially the model of Gimeno et al. The combination of this strategy with educational and rescue strategies may enhance bowel cleansing and reduce the need for repeated procedures.

## Figures and Tables

**Table 1:** Indications for colonoscopy.

Indication	N (%)
Average/family risk CRC screening	215 (33.1)
Postpolypectomy/CRC surveillance	180 (27.7)
Inflammatory bowel disease	57 (8.8)
Change in bowel habits	55 (8.4)
Hematochezia	45 (6.9)
Anemia	45 (6.9)
Others	52 (8.1)

**Table 2:** Comparison of accuracy among models.

	Difference in AUC	SE	95% CI	*p*
Model 1 vs. 2	0.0499	0.0202	0.0103–0.0895	0.013
Model 1 vs. 3	0.132	0.0296	0.0741–0.190	<0.001
Model 2 vs. 3	0.0822	0.0278	0.0278–0.137	0.003
New model vs. 1	0.00347	0.0142	−0.0244–0.0314	0.807
New model vs. 2	0.0534	0.0204	0.0134–0.0935	0.009
New model vs. 3	0.136	0.0317	0.0736–0.198	0.013

Abbreviations: AUC: area under the curve; SE: standard error; CI: confidence interval.

**Table 3:** Multiple logistic regression analysis of risk factors associated with inadequate bowel cleansing.

Risk Factors	OR * (95% CI **)	Wald Coefficient	*p*
Renal failure	4.429 (1.237–15.857)	5.229	0.022
ECOG †	2.884 (1.431–5.813)	8.772	0.003
Diabetes mellitus	2.494 (1.568–3.967)	14.901	<0.001
Constipation	2.370 (1.476–3.807)	12.748	<0.001

* OR: odds ratio; ** CI: confidence interval; † ECOG: Eastern Cooperative Oncology Group.

**Table 4:** Performance of the new predictive model.

	Global BBPS †	Proximal Colon BBPS	Transverse Colon BBPS	Left Colon BBPS
Sensitivity	68/102 (66.7%)	61/90 (67.8%)	48/68 (70.6%)	38/59 (64.4%)
Specificity	344/547 (62.9%)	345/553 (62.4%)	358/581 (61.6%)	357/590 (60.5%)
PPV *	68/271 (25.1%)	61/269 (21.7%)	48/271 (17.7%)	38/271 (14%)
NPV **	344/378 (91%)	345/374 (92.2%)	358/378 (94.7%)	357/378 (94.4%)

Positive likelihood ratio = 1.796, 95% CI [1.508–2.141]; negative likelihood ratio = 0.53, 95% CI [0.4–0.703]; * PPV: positive predictive value; ** NPV: negative predictive value; † BBPS: Boston Bowel Preparation Scale.

## Data Availability

Research data can be provided by the authors.

## Supplementary Materials

The following supporting information can be downloaded at: https://www.mdpi.com/article/10.3390/jpm14010102/s1, Supplementary Materials file S1: Current predictive models in outpatients.; Table S1: Comparison of patients with and without risk factors for poor bowel cleansing on univariate analysis; Table S2: Wald coefficients and score for each independent variable.

## Abbreviations

Colorectal cancer: CRC; Boston Bowel Preparation Scale: BBPS; Area under the ROC curve: AUC; confidence interval: CI; positive predictive value: PPV; negative predictive value: NPV; positive likelihood ratio: LR+; negative likelihood ratio: LR−; Eastern Cooperative Oncology Group: ECOG; American Society of Anesthesiologists: ASA; polyethylene glycol: PEG; ascorbic acid: Asc.

## References

[1] Brenner H., Stock C., Hoffmeister M. (2014). Effect of screening sigmoidoscopy and screening colonoscopy on colorectal cancer incidence and mortality: Systematic review and meta-analysis of randomised controlled trials and observational studies. BMJ.

[2] Kaminski M.F., Thomas-Gibson S., Bugajski M., Bretthauer M., Rees C.J., Dekker E., Hoff G., Jover R., Suchanek S., Ferlitsch M. (2017). Performance measures for lower gastrointestinal endoscopy: A European Society of Gastrointestinal Endoscopy (ESGE) Quality Improvement Initiative. Endoscopy.

[3] Robertson D.J., Lee J.K., Boland C.R., Dominitz J.A., Giardiello F.M., Johnson D.A., Kaltenbach T., Lieberman D., Levin T.R., Rex D.K. (2017). Recommendations on Fecal Immunochemical Testing to Screen for Colorectal Neoplasia: A Consensus Statement by the US Multi-Society Task Force on Colorectal Cancer. Gastroenterology.

[4] Hassan C., East J., Radaelli F., Spada C., Benamouzig R., Bisschops R., Bretthauer M., Dekker E., Dinis-Ribeiro M., Ferlitsch M. (2019). Bowel preparation for colonoscopy: European Society of Gastrointestinal Endoscopy (ESGE) Guideline—Update 2019. Endoscopy.

[5] Adams W.J., Meagher A.P., Lubowski D.Z., King D.W. (1994). Bisacodyl reduces the volume of polyethylene glycol solution required for bowel preparation. Dis. Colon. Rectum.

[6] Hassan C., Fuccio L., Bruno M., Pagano N., Spada C., Carrara S., Giordanino C., Rondonotti E., Curcio G., Dulbecco P. (2012). A predictive model identifies patients most likely to have inadequate bowel preparation for colonoscopy. Clin. Gastroenterol. Hepatol..

[7] Berger A., Cesbron-Metivier E., Bertrais S., Olivier A., Becq A., Boursier J., Lannes A., Luet D., Pateu E., Dib N. (2021). A predictive score of inadequate bowel preparation based on a self-administered questionnaire: PREPA-CO. Clin. Res. Hepatol. Gastroenterol..

[8] Dik V.K., Moons L.M., Huyuk M., van der Schaar P., de Vos Tot Nederveen Cappel W.H., Ter Borg P.C., Meijssen M.A., Ouwendijk R.J., Le Fevre D.M., Stouten M. (2015). Predicting inadequate bowel preparation for colonoscopy in participants receiving split-dose bowel preparation: Development and validation of a prediction score. Gastrointest. Endosc..

[9] Gimeno-Garcia A.Z., Baute J.L., Hernandez G., Morales D., Gonzalez-Pérez C.D., Nicolás-Pérez D., Alarcon-Fernández O., Ji-ménez A., Hernandez-Guerra M., Romero R. (2017). Risk factors for inadequate bowel preparation: A validated predictive score. Endoscopy.

[10] Johnson D.A., Barkun A.N., Cohen L.B., Dominitz J.A., Kaltenbach T., Martel M., Robertson D.J., Boland C.R., Giardello F.M., Lieberman D.A. (2014). Optimizing adequacy of bowel cleansing for colonoscopy: Recommendations from the US multi-society task force on colorectal cancer. Gastroenterology.

[11] Saltzman J.R., Cash B.D., Pasha S.F., Early D.S., Muthusamy V.R., Khashab M.A., Chathadi K.V., Fanelli R.D., Chandrasekhara V., Lightdale J.R. (2015). Bowel preparation before colonoscopy. Gastrointest. Endosc..

[12] Lai E.J., Calderwood A.H., Doros G., Fix O.K., Jacobson B.C. (2009). The Boston bowel preparation scale: A valid and reliable instrument for colonoscopy-oriented research. Gastrointest. Endosc..

[13] Afecto E., Ponte A., Fernandes S., Gomes C., Correia J.P., Carvalho J. (2023). Validation and Application of Predictive Models for Inadequate Bowel Preparation in Colonoscopies in a Tertiary Hospital Population. GE Port. J. Gastroenterol..

[14] Ness R.M., Manam R., Hoen H., Chalasani N. (2001). Predictors of inadequate bowel preparation for colonoscopy. Am. J. Gastroenterol..

[15] Guo X., Yang Z., Zhao L., Leung F., Luo H., Kang X., Li X., Jia H., Yang S., Tao Q. (2017). Enhanced instructions improve the quality of bowel preparation for colonoscopy: A meta-analysis of randomized controlled trials. Gastrointest. Endosc..

[16] Gimeno-García A.Z., Hernández-Pérez A., Nicolás-Pérez D., Hernández-Guerra M. (2023). Artificial Intelligence Applied to Colonoscopy: Is It Time to Take a Step Forward?. Cancers.

[17] Ibanez-Sanz G., Sanz-Pamplona R., Garcia M. (2022). Post-polypectomy colonoscopy surveillance: Can we improve the diagnostic yield?. Gastroenterol. Hepatol..

[18] Machlab S., Martinez-Bauer E., Lopez P., Piqué N., Puig-Diví V., Junquera F., Lira A., Brullet E., Selva A., García-Iglesias P. (2021). Comparable quality of bowel preparation with single-day versus three-day low-residue diet: Randomized controlled trial. Dig. Endosc..

