# Supplementary material for: Comparative Study of Predictive Models for the Detection of Patients at High Risk of Inadequate Colonic Cleansing
Source: J Pers Med. 2024 Jan 17;14(1):102. doi: 10.3390/jpm14010102 (PMC10820399; doi:10.3390/jpm14010102)
Supplement: Supplementary file 1 [file jpm-14-00102-s001.zip › Supplementary Materials file S1.pdf]

Supplementary Materials file S1. Current predictive models in outpatients.

	***Dik* et al.**	***Gimeno* et al.**	***Berger* et al.**
Variables	-ASA score<br>-Tricyclic antidepressants<br>-Opioids<br>-Diabetes<br>-Chronic constipation<br>-Abdominal/pelvic surgery<br>-Hospitalization<br>-History of inadequate preparation	-Tricyclic antidepressants<br>-Comorbidities<br>-Chronic constipation<br>-Abdominal/pelvic surgery	-Diabetes/obesity<br>-Irregular physical activity<br>-Cirrhosis<br>-Antidepressants/neuroleptics<br>-Opioids<br>-Abdominal surgery<br>-History of inadequate preparation
AUC, 95% CI	0.72-0.77*	0.72-0.70*	0.622-0.621*

* AUC in the development and validation cohorts.
