# Supplementary material for: Comparative Study of Predictive Models for the Detection of Patients at High Risk of Inadequate Colonic Cleansing
Source: J Pers Med. 2024 Jan 17;14(1):102. doi: 10.3390/jpm14010102 (PMC10820399; doi:10.3390/jpm14010102)
Supplement: Supplementary file 1 [file jpm-14-00102-s001.zip › Supplementary Table S1.pdf]

Supplementary table S1. Comparison of patients with and without risk factors for poor bowel cleansing on univariate analysis

Risk factor	Adequate bowel cleansing (n=547)	Poor bowel cleansing (n=102)	OR* (95% CI**)	P
Age (mean±SD†)	60.44±12.956	64.13±12.08	1.024 (1.006-1.042)	0.008
Sex, n (%)			1.24 (0.81-1.90)	0.31
Male	276 (50.5)	57 (55.9)		
Female	271 (49.5)	45 (44.1)		
BMI‡, kg/m2, (mean±SD)	27.925±4.954	28.579±5.901	1.025 (0.984-1.067)	0.238
Comorbidity, n (%)			3.534 (2.279-5.480)	<0.001
Yes	117 (21.4)	50 (49.0)		
No	430 (78.6)	52 (51)		
Diabetes mellitus, n (%)			3.048 (1.955-4.754)	<0.001
Yes	109 (19.9)	44 (43.1)		
No	438 (80.1)	58 (56.9)		
Cirrhosis, n (%)			5.406 (0.335-87.132)	0,18
Yes	1 (0.2)	1 (1.0)		
No	546 (99.8)	101 (99.0)		

Renal failure, n (%)<br><br>Yes<br><br>No	 <br>6 (1.1)<br><br>541 (98.9)	 <br>5 (4.9)<br><br>97 (95.1)	4.648 (1.391-15.529)	0.006
Stroke<br><br>Yes<br><br>No	 <br>9 (1.6)<br><br>538 (98.4)	 <br>6 (5.9)<br><br>96 (94.1)	3.736 (1.300-10.736)	0.02
ASA<br><br>1<br><br>2<br><br>3	 <br>193 (35.5)<br><br>315 (57.9)<br><br>36 (6.6)	 <br>27 (26.5)<br><br>69 (67.6)<br><br>6 (5.9)	 <br><br><br>1.472 (0.928-2.335)<br><br>1.072 (0.416-2.761)	0.24
Opioids, n (%)<br><br>Yes<br><br>No	 <br>9 (1.6)<br><br>538 (98.7)	 <br>6 (5.9)<br><br>96 (94.1)	3.736 (1.3-10.736)	0.009
Antidepressants, n (%)<br><br>Yes<br><br>No	 <br>35 (6.4)<br><br>512 (93.6)	 <br>13 (12.7)<br><br>89 (87.3)	1.939 (0.842-4.464)	0.114

Neuroleptics, n (%)<br>Yes<br>No	 23 (4.2)<br>524 (95.8)	 8 (7.8)<br>94 (92.2)	2.948 (1.345-6.463)	0.005
Constipation, n (%)<br>Yes<br>No	 106 (19.4)<br>441 (80.6)	 38 (37.3)<br>64 (62.7)	2.470 (1.569-3.889)	<0.001
Family history of Colorectal cancer, n (%)<br>Yes<br>No	 146 (26.7)<br>401 (73.3)	 16 (15.7)<br>86 (84.3)	0.511 (0.29-0.9)	0.018
Low educational level, n (%)<br>Yes<br>No	 255 (46.7)<br>291 (53.3)	 63 (61.8)<br>39 (38.2)	1.845 (1.194-2.841)	0.005
Abdominal/pelvic surgery, n (%)<br>Yes<br>No	 178 (32.5)<br>369 (67.5)	 35 (34.3)<br>67 (65.7)	1.083 (0.693-1.692)	0.726

Type of preparation, n (%)				0.43
PEG# 4 L (reference category)	46 (8.4)	9 (8.8)		
PEG 2 L + AA	289 (52.8)	59 (57.8)	1.015 (0.487-2.118)	
SP¶	120 (21.9)	22 (21.6)	0.874(0.387-1.978)	
PEG 1 L + AA	92 (16.8)	12 (11.8)	0.587(0.236-1.459)	
Regular exercise, n (%)			1.219 (0.797-1.865)	0.360
Yes	225 (41.2)	47 (46.1)		
No	321 (58.8)	55 (53.9)		
ECOG§, n (%)			3.183 (1.634-6.203)	0.001
0-1	517 (94.9)	87 (85.3)		
≥2	28 (5.1)	15 (14.7)		
Diet adherence, n (%)			0.510 (0.179-1.447)	0.198
Yes	533 (97.4)	97 (95.1)		
No	14 (2.6)	5 (4.9)		
Vomiting, n (%)			0.903 (0.519-1.572)	0.719
Yes	390 (80.1)	69 (78.4)		
No	97 (19.9)	19 (21.6)		

*OR: odds ratio; **CI: confidence interval; †SD: standard deviation; ‡BMI: body mass index; *PEG: polyethylene glycol plus ascorbic acid; #AA: ascorbic acid; ¶SP: sodium picosulfate plus magnesium citrate plus citric acid; §ECOG: Eastern Cooperative Oncology Group.
