# Supplementary material for: Comparative Study of Predictive Models for the Detection of Patients at High Risk of Inadequate Colonic Cleansing
Source: J Pers Med. 2024 Jan 17;14(1):102. doi: 10.3390/jpm14010102 (PMC10820399; doi:10.3390/jpm14010102)
Supplement: Supplementary file 1 [file jpm-14-00102-s001.zip › Supplementary Table S2.pdf]

Supplementary table S2. Wald coefficients and score for each independent variable

**Risk factors**	**Wald coefficient**	***Score***
Diabetes mellitus	14.910	4
Constipation	12.748	3.420
ECOG *	8.772	2.353
Renal failure	5.229	1.403

*ECOG: Eastern Cooperative Oncology Group.
